# Artificial Intelligence-Based MRI Segmentation for the Differential Diagnosis of Single Brain Metastasis and Glioblastoma

**DOI:** 10.3390/diagnostics15172248

**Published:** 2025-09-05

**Authors:** Daniela Pomohaci, Emilia-Adriana Marciuc, Bogdan-Ionuț Dobrovăț, Mihaela-Roxana Popescu, Ana-Cristina Istrate, Oriana-Maria Onicescu (Oniciuc), Sabina-Ioana Chirica, Costin Chirica, Danisia Haba

**Affiliations:** 1 Doctoral School, Grigore T. Popa University of Medicine and Pharmacy, 16 Universității Str., 700115 Iasi, Romania; danielapomohaci@yahoo.com (D.P.); anacristinaistrate@yahoo.com (A.-C.I.); chiricasabinaioana@gmail.com (S.-I.C.); 2 Department of Oral and Maxillofacial Surgery, Faculty of Dental Medicine, Grigore T. Popa University of Medicine and Pharmacy, 16 Universității Str., 700115 Iasi, Romania; emma.marciuc@gmail.com (E.-A.M.); bogdan.dobrovat@yahoo.com (B.-I.D.); drpopescuroxana@yahoo.com (M.-R.P.); danihaba@yahoo.com (D.H.); 3 Department of Radiology, Emergency Hospital Professor Doctor Nicolae Oblu, 700309 Iasi, Romania; 4 Faculty of Computer Science, “Alexandru Ioan Cuza” University, 700506 Iasi, Romania; oriana.oniciuc@info.uaic.ro

**Keywords:** glioblastoma, brain metastasis, artificial intelligence, deep learning, segmentation, differential diagnosis

## Abstract

**Background/Objectives**: Glioblastomas (GBMs) and brain metastases (BMs) are both frequent brain lesions. Distinguishing between them is crucial for suitable therapeutic and follow-up decisions, but this distinction is difficult to achieve, as it includes clinical, radiological and histopathological correlation. However, non-invasive AI examination of conventional and advanced MRI techniques can overcome this issue. **Methods**: We retrospectively selected 78 patients with confirmed GBM (39) and single BM (39), with conventional MRI investigations, consisting of T2W FLAIR and CE T1W acquisitions. The MRI images (DICOM) were evaluated by an AI segmentation tool, comparatively evaluating tumor heterogeneity and peripheral edema. **Results**: We found that GBMs are less edematous than BMs (*p* = 0.04) but have more internal necrosis (*p* = 0.002). Of the BM primary cancer molecular subtypes, NSCCL showed the highest grade of edema (*p* = 0.01). Compared with the ellipsoidal method of volume calculation, the AI machine obtained greater values when measuring lesions of the occipital and temporal lobes (*p* = 0.01). **Conclusions**: Although extremely useful in radiomics analysis, automated segmentation applied alone could effectively differentiate GBM and BM on a conventional MRI, calculating the ratio between their variable components (solid, necrotic and peripheral edema). Other studies applied to a broader set of participants are necessary to further evaluate the efficacy of automated segmentation.

## 1. Introduction

Glioblastomas (GBM) and brain metastases (BMs) are the most frequent tumoral pathologies of the brain parenchyma [1,2]. GBM is a grade 4 glioma in the World Health Organization classification of Central Nervous System tumors, representing 40–50% of primary brain tumors in adult patients [3,4,5]. BMs on the other hand are the most common secondary malignancies in adult patients, being 10 times more frequent than brain parenchyma primary tumors [2]. Secondary lesions affecting the brain parenchyma are in 30–50% of cases a solitary BM [6,7].

The differential diagnosis between solitary BM and GBM is important as it plays a pivotal role in determining the prognosis, treatment and the monitoring phase of the diseases [6,8]. For solitary metastases, if their diameter is greater than 3 cm, surgical resection of the entire lesion is preferred, and if the diameter is less than 3 cm, stereotactic radiosurgery is opted for. Target therapy is also an option of treatment for patients with a BM of known origin and known molecular subtype of the primary tumor [9]. For GBM, the debulking of the lesion is chosen, as incomplete resection can decrease the burden of an intra-cranial expansive process and ameliorate the patients’ symptoms [5,6,10].

Regardless of their different natures, being either primary or secondary tumors, they are both classified as intra-cerebral expansive processes [11]. Focal symptoms caused by the tumor itself and intra-cranial hypertension caused by both the lesion and the peripheral edema are the most commonly observed symptoms among patients.

In case of metastatic disease of the brain, if the primary tumor is found or systemic metastases are also present, the diagnosis can be easily concluded as concomitant GBM, and systemic neoplasia is quite rare, found in 3% of cases [12]. Patients with a solitary BM from an occult primary cancer can be difficult to correctly diagnose solely based on the imaging characteristics of the lesion, as the diagnostic process in both cases requires correlation with clinical and histopathological findings [8].

The aspects of brain primary and secondary lesions, using both Computed Tomography (CT) and Magnetic Resonance Imaging (MRI) can be misleading, as GBMs and BMs share similar characteristics [8]. On CT they both are inhomogeneous masses of different volumes accompanied by hypodense peripheral edema [13]. On conventional MRI protocols, they both are inhomogeneous lesions, with central necrosis, predominantly peripheral contrast intake and perilesional vascular edema [14]. BMs, more frequently than GBMs, can show cystic degeneration and intra-lesional hemorrhage (especially hemorrhagic metastases of melanoma and renal cancer). GBM on the other hand, frequently shows invasion of the surrounding brain parenchyma, seen on MRI similar to peripheral edema [14].

A definitive diagnosis is generally established through a biopsy of the lesion or after surgical resection [8]. As these interventions have their own complications and because surgical resection is not always possible, as in the case of eloquent brain tissue being affected, another non-invasive method of diagnosis should be adopted [8].

Since the conventional MRI features of both GBMs and BMs are similar, displaying perilesional edema, central necrosis, irregular margins and inhomogeneous contrast intake, differentiating based on MRI imaging alone can be difficult [8,15]. Advanced MRI modalities have therefore emerged, such as perfusion MRI, diffusion tensor imaging (DTI), diffusion-weighted imaging (DWI) and magnetic resonance spectroscopy, combined with radiomics or not [6,8,10,15,16,17].

As shown by previous studies, the addition of advanced MRI protocols, as input on machine learning (ML) or deep-learning (DL) programs, provides no further insight than the use of only conventional MRI protocols [18]. One particularly important step in radiomics analysis of the extracted features is tumoral segmentation or the selection of the Region of Interest (ROI). This step was previously executed manually by neuro-radiologists; now semi-automated and automated segmentation can be performed by artificial intelligence (AI) programs [19]. Automated segmentation can be realized by combining two or more MRI acquisition protocols, in order to visualize different portions of the lesions, as they are inhomogeneous and have different signal capacities depending on their composition [13,18].

Our scope is to analyze the different ratios of tumoral volume, peripheral edema and necrosis volumes of GBM and unique BM, calculated using an automated segmentation model, in order to individualize possible significant differences in their values, with further clinical implications in diagnosis and suitable therapeutical protocol choice. Another objective of this study is to compare the automated segmentation model to the ellipsoidal manual method of tumoral volume calculation. This method has been previously described as one of the most accurate manual methods of volume calculation [19].

## 2. Materials and Methods

### 2.1. Patient Selection

We retrospectively selected patients diagnosed with GBM and BM in our hospital from 2019 to 2023. The inclusion criteria were as follows:

1. Adult patients (more than or equal to 18 years old in age);
2. Those diagnosed with a unique brain lesion;
3. Those with a histopathological positive diagnosis of GBM or BM;
4. Those who underwent MRI examination with a protocol consisting at minimum of 3D T2-weighted Fluid-Attenuated Inversion Recovery (T2W FLAIR) and contrast-enhanced T1-weighted (CE T1W) effectuated prior to any local treatment.

We excluded patients who did not meet the inclusion criteria and who also:

1. Had more than one lesion in the brain parenchyma, active or not, for example, with stroke sequelae;
2. Did not have any histopathological confirmation;
3. Underwent MRI investigation after local treatment or biopsy.

### 2.2. MRI Protocols

The patients underwent MRI examinations during their hospital stay, executed with a General Electric Medical Systems—Signa explorer MRI machine of 1.5 Tesla. The MRI protocol consisted of sagittal 3D T2 FLAIR with acquisition parameters of repetition time (TR) = 6502–7002, echo time (TE) = 118.597–122.618, slice thickness of 1.8 mm and spacing between slices of 0.9 mm; 3D Fast Spoiled Gradient Echo (FSPGR) T1W after contrast intake, with acquisition parameters of TR = 19.892 and TE = 4.2 or 3D T1W after contrast intake, with TR = 9.464 and TE = 4.2, the latter two both with slice thicknesses of 1.8 mm and spacing between slices of 0.9 mm. The difference in the TR value is normal for FSPGR T1W and non-FSPGR T1W; the first acquisition is usually preferred after contrast intake because the vascular signal is lower, and small lesions can be easily seen [20]. The images were anonymized and then analyzed using the AI software.

### 2.3. Manual Calculation of Tumoral Volume

The manual method that we used to calculate the volume of the lesions was the ellipsoidal method. The first step consists in correctly positioning the T1W 3D CE acquisition in the axial, sagittal and coronal planes, by orienting the medio-sagittal line in the coronal and axial planes and the bi-commissural axis (the line that conjoins the anterior and posterior white matter commissures) in the sagittal plane. The second step represents measuring the lesion by assessing the greater diameter in the three planes and multiplying them to obtain the volume. Because the resulting shape is more similar to a parallelepiped or a cube, it is necessary to multiply the result with the ellipsoidal constant of 0.52 (π/6), which will consequently change the final shape to resemble an ellipse. This shape is found to be closer to the true shape of brain lesions [19]. The total volume of the lesion was calculated initially using the ellipsoidal manual method by two different neuro-radiologists, each with a minimum of 10 years of experience in the field.

### 2.4. Automated Segmentation

The images were also sent for the automated segmentation of the tumor, with calculation of the total tumor volume, consisting of the contrast-intake portion (solid component of the lesion) and of the central necrosis volume (non-enhancing portion of the lesion), as well as the peripheral edema volume.

**Figure 1:**
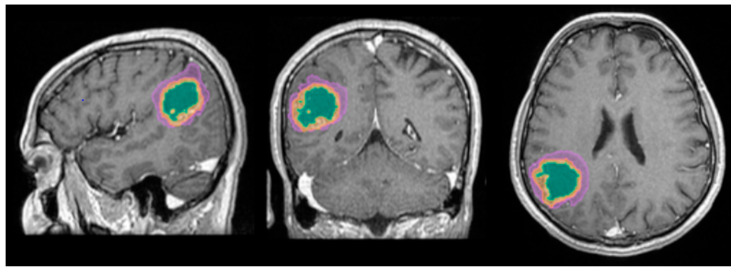
Mdbrain processing of MRI brain acquisition (DICOM) (Scale 1:20). T1W CE in sagittal, coronal and axial planes, respectively. View of the three regions that were calculated separately for every patient: green—necrosis, orange—enhancing part of the tumor and purple—perilesional edema (approval was obtained from the Ethics Committee of the University of Medicine and Pharmacy “Grigore T. Popa” Iasi).

The automated segmentation was implemented using the mdbrain application, certified as a Conformité Européenne medical device of II class, developed by the German company of Mediaire, available in Romania through Supermedical. Mdbrain uses a DL algorithm consisting of a 3D convolutional neural network with a U-Net architecture [21]. The T2-weighted Fluid-Attenuated Inversion Recovery (T2W FLAIR) and T1-weighted contrast-enhanced (CE T1W) images were priorly combined in order to see the tumor and the edema on the same image, as the tumor enhances on the CE T1W images, and the edema shows a hyperintense signal on FLAIR and does not enhance [22]. The results of the Digital Imaging and Communication in Medicine (DICOM) imaging processing tool are seen in Figure 1.

### 2.5. Database Creation

We created an Excel table containing data about the patients: age, sex and diagnosis: primary tumor (in the case of BM) and GBM; we then added data about the solitary lesions for every patient: volume calculated by the ellipsoid manual method and by the automated method, with an emphasis on the total volume, the contrast-intake portion, the non-enhancing portion and the peripheral edema volume. A comparison of the two methods was performed by calculating the ratio and difference between the respective total volumes using Excel formulas; we determined the ratio between the automated calculated volumes of the necrosis (non-enhancing portion) and the total volume, between the edema and the total volume and separately with the necrosis in order to appreciate the percentage of necrosis within the tumors and the time-fold dimension of the edema compared with the lesion. An example of the formula used can be seen below:

(1) $$R=\frac{Vn}{Vt}$$

where *R*—ratio, *Vn*—volume of the necrosis and *Vt*—total volume; *Vn* and *Vt* are both calculated by the automated segmentation method. Similarly, if we switch *Vn* with *Vt* and *Vt* with *Ve* (volume of edema), we obtain the ratio between them.

In order to avoid any confusion and to minimize the repetition of these rapports, we annotated them as follows:

1. *R* _AI/M_—the ratio between the volumes obtained using the DL tool (AI) and the ellipsoidal method (the manual method—M), respectively;
2. *R* _E/T_—the ratio between the volumes of the edema (E) and the lesion (T) calculated using the DL software;
3. *R* _N/T_—the ratio between the volumes of the necrosis (N) and the entire lesion (T) calculated using the Dl software;
4. *R* _E/N_—the ratio between the volumes of the edema (E) and the central necrosis (N) calculated using the DL software;
5. Diff _AI-M_—the difference between the volumes obtained by the AI software and the manual method (M).

### 2.6. Statistical Analysis

**Figure 2:**
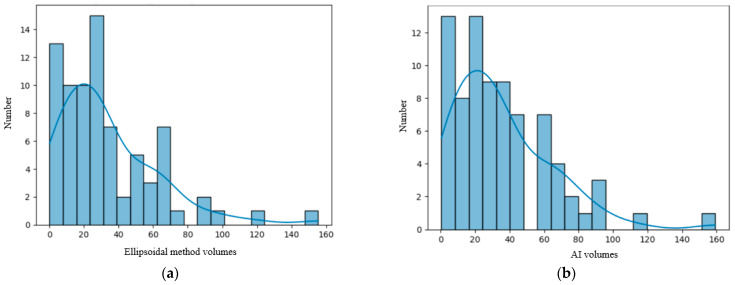

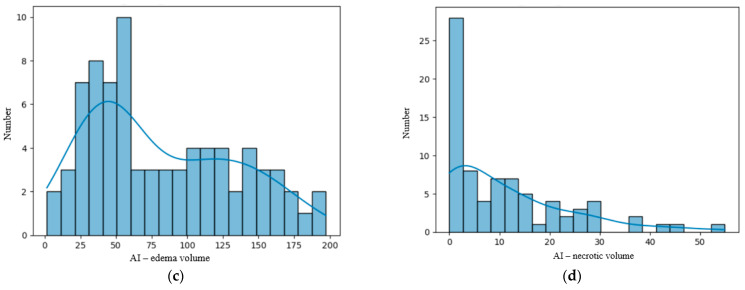
(**a**) Distribution of the volume of the lesions calculated using the ellipsoidal method. (**b**) Distribution of the lesions’ volume calculated by the AI tool. (**c**) Distribution of the perilesional edema volume of the lesions calculated using the AI tool. (**d**) Distribution of the necrosis volume calculated by the AI tool.

**Figure 3:**
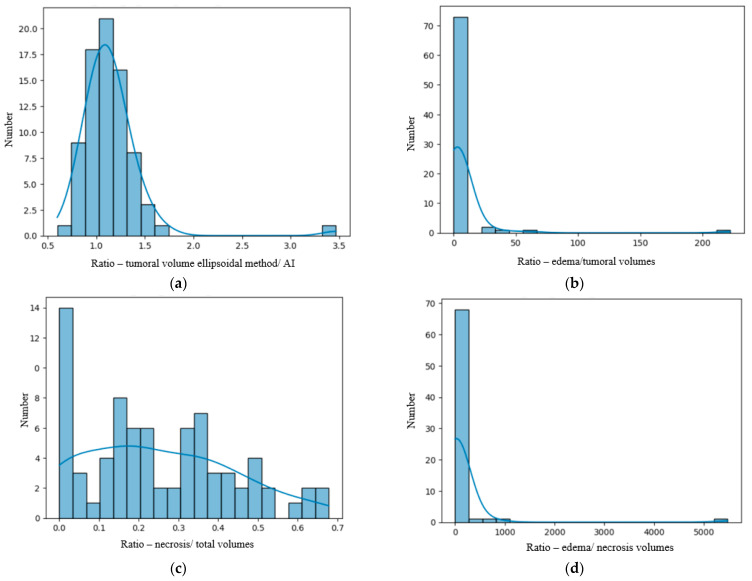
(**a**) Distribution of the *R* _AI/M_ (ratio between the tumoral volumes obtained by the AI method and the manual method). (**b**) Distribution of the *R*_E/T_ (ratio between edema and total tumoral volumes) calculated by the AI tool. (**c**) Distribution of the *R*_N/T_ (ratio between the central necrosis and total tumoral volumes) calculated using the AI tool. (**d**) Distribution of the *R* _E/N_ (ratio between the edema and necrosis volumes).

We analyzed our data distribution using the Kolmogolov–Smirnov test, in order to apply the suitable parametric or non-parametric tests for Gaussian and non-Gaussian distribution variables; see Figure 2 and Figure 3 [23]. We also performed bivariate analysis to show the relation between two different binary and numerical attributes, generating box plots. In order to analyze the significance of the volume ratios for different groups of variables, we used the ANOVA one-way test in the multivariate analysis.

#### 2.6.1. Univariate Analysis

The numerical variables examined included age, lesion volume and the ratios of the volumes. As seen in Figure 2 and Figure 3, they had a non-Gaussian distribution. Diff _AI-M_ had a Gaussian distribution (see the figure in Section 3.3).

#### 2.6.2. Bivariate Analysis

We then examined the relation between each pair of variables. To identify differences between subgroups defined by the binary attributes and continuous values, we used statistical tests and generated box plots. The volume and the ratios had a non-Gaussian distribution; therefore, we used non-parametric tests such as the Mann–Whitney-U test. However, for age and Diff _AI-M_, we used a parametric test, the Student *t*-test, considering the value of *p* ≤ 0.05 statistically significant [23,24].

#### 2.6.3. Multivariate Analysis

We also assessed the statistical significance of the variation of variables for more than two groups of data using the ANOVA one-way test for variables with a Gaussian distribution (Diff _AI-M_) and the analog non-parametric test, the Kruskal test, for variables with a non-Gaussian distribution [25].

### 2.7. Processing Environment

The statistical analysis was performed using the Python 3.13.2 version and packages from different code libraries such as “pandas”, “seaborn”, “numpy”, “matplotlib” and “scipy”.

## 3. Results

In total, we found 578 patients admitted at our hospital with the diagnosis of BM and 695 patients with GBM, during a period of five years (2019–2023). Of the ones with BMs, only 178 had a positive histological and immunopathological diagnosis, and only 109 underwent an MRI investigation prior to any local treatment, of which 58 had a singular brain lesion. Only 39 were included in the study, as many of them showed other brain lesions.

In total, 85 patients were diagnosed with GBM and underwent histopathological confirmation and MR investigation; 39 of them were selected to be analyzed with the AI program. We did not include patients with other concomitant brain lesions, as discussed in the Materials and Methods Section. In total, we included 78 participants: 54 male patients and 24 female patients.

### 3.1. Cohort Description

#### 3.1.1. Glioblastoma Patients

The GBM patients included in this study were 39; 28 of them were men and 11 were women, ranging from 36 to 76 years old, with a median age of 53.89 years old. The characteristics of the patients can be seen in Table 1.

#### 3.1.2. Single Brain Metastasis Patients

For the BM subgroup, 26 were men and 13 were women, with ages between 21 and 78 years old, and a median age of 58.89 years old. Their primary tumors were known, with 24 patients diagnosed with lung cancer, 7 with breast cancer, 3 with melanoma, 2 with colorectal carcinoma, 2 patients with other digestive system sites (of which one was diagnosed with gastric carcinoma) and 1 with clear-cell renal carcinoma. The characteristics of the patients can be seen in Table 1 and the molecular subtype of the primary tumors in Table 2.

### 3.2. Primary Results

**Figure 4:**
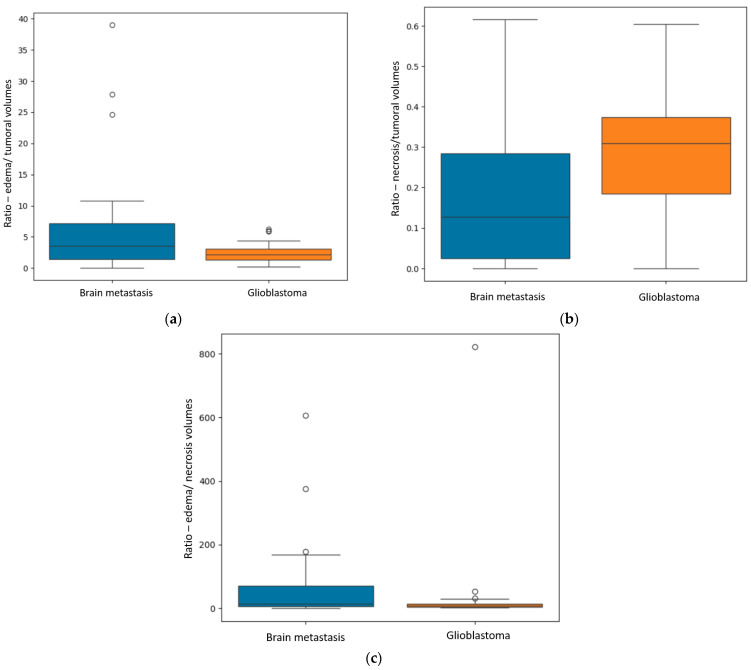
(**a**) Box plot showing the *R* _E/T_ (ratio between the edema and the central necrosis volumes) for the two subgroups. (**b**) Box plot displaying the difference in the *R*_N/T_ (ratio between the necrosis and total tumoral volumes). (**c**) Box plot representing the difference in the *R* _E/N_ (ratio between the edema and necrosis volumes) for the two subgroups.

Significant findings were achieved after the statistical analysis of the data, particularly regarding the difference in the ratios between the different volumes for GBM and secondary brain lesions, as shown in Figure 4.

The first finding was the statistically significant difference between the *R* _E/T_ for secondary brain lesions and GBM (*p* = 0.04—Mann–Whitney-U test), with significantly greater values for the metastases: a median of 3.59 with a minimum of 0.06 and a maximum of 10.81, and smaller values for the GBM group: a median of 2.2 with a minimum of 0.25 and a maximum of 4.4.

The second primary result was the difference in the *R*_N/T_ (*p* = 0.002—Mann–Whitney-U test), with grater values for the GBM subpopulation, as follows: a median of 0.3, minimum of 0 (no necrotic center) and maximum of 0.6. For the BM subgroup, the median value was 0.12, with a minimum of 0 (no central necrosis) and a maximum of 0.61.

Another significant finding was the *R*_E/N_ (*p* = 0.02), with greater values for the metastatic subgroup, suggesting greater inconsistencies between perilesional edema and central necrosis (greater edema and lesser necrosis). The median value for the metastatic group was 70.6, with a minimum of 0.09 and a maximum of 167.84; the GBM subgroup had a median value of 28.52, with a minimum of 0 (no central necrosis) and maximum of 53.15.

### 3.3. Secondary Results

**Figure 5:**
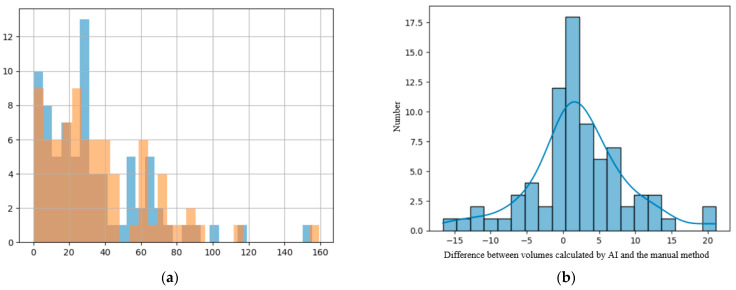
(**a**) Histogram showing the superposition of the manual method (blue columns) and the DL method (orange columns); *x*-axis—value of the volume and *y*-axis—number of lesions with that volume. (**b**) Distribution of difference between volumes calculated with the manual method and the DL method.

Comparing the two different modalities used to calculate the volume of the lesions, we found that their values are predominantly superposable and that the numerical difference between them had a normal distribution, as the resulting values are volumes measured in cm^3^, closely resembling the standard normal distribution, as seen in Figure 5b [23].

**Figure 6:**
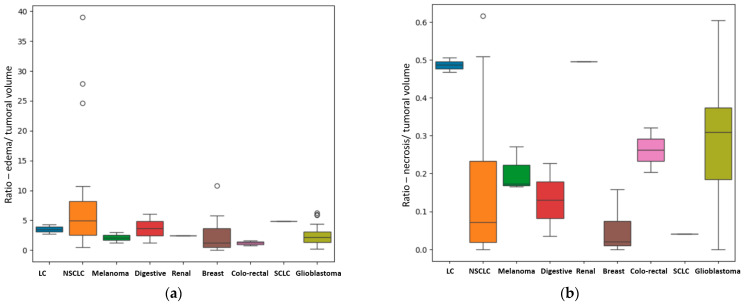
(**a**) Box plot showing the different values of the median, minimum and maximal values for the *R* _E/T_, according to their primary tumor (if secondary lesions) and GBM. In this box plot, the different molecular subtypes of lung cancer are separately assessed: the NSCLC subgroup has the more edematous lesions, with external values close to 40; the SCLC subgroup consisting of only one patient has a significantly smaller *R*_E/T_ and the LC subgroup—unknown molecular subtype has even lower values (close to 4). (**b**) Box plot showing the different values of the median, minimum and maximal values of *R* _N/T_, according to their primary tumor (if secondary lesions) and GBM. In this box plot, the different molecular subtypes of lung cancer are separately assessed with the LC subgroup—unknown molecular subtype showing the greatest central necrosis in the LC group. GBM is the most necrotic lesion, with a median value of 0.31 and with a maximum ratio of 0.6.

For the patients with a unique BM, other results were also of statistical importance regarding in particular the primary source. We assessed the difference in metastatic volume for every different primary cancer, compared with GBM, using the ANOVA test for non-parametric distribution (Kruskal test). We found a *p*-value of 0.01 that indicates statistical importance regarding the *R*_E/T_ of the lesions (Figure 6a) with the most edematous lesions being the BM from lung cancer, and in particular from NSCLC, followed by the ones from digestive carcinomas (including gastric carcinoma), from breast cancer, melanoma, renal carcinoma, GBM (as a primary tumor) and colorectal carcinomas. The *p*-value was calculated after considering the patients with BM from lung cancer (LC) as a single subgroup (LC + NCLSC + SCLC); when considering them separate as determined by their molecular subtype, the calculated *p*-value was 0.03.

Another important finding is the difference in terms of the quantity of central necrosis, with a *p*-value of 0.01 for the *R* _N/T_ (Figure 6b). Our findings strongly suggest that GBM, as a primary brain lesion, has considerably more central necrosis than secondary lesions, of any kind, the most necrotic ones being the BMs from colorectal and renal carcinomas. When considering the LC patients as separate groups depending on their molecular subtype, the calculated *p*-value was 0.001.

**Figure 7:**
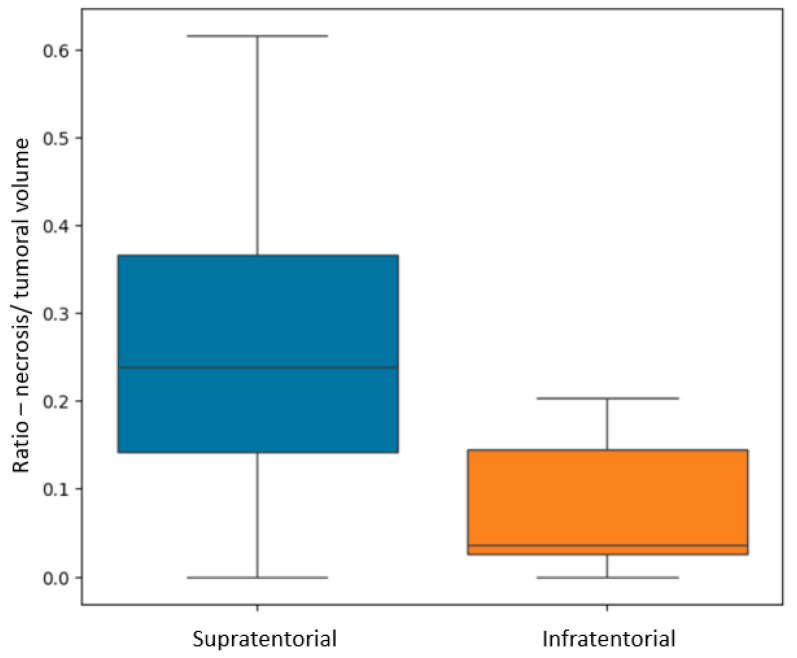
Box plot showing the difference in *R* _N/T_ (ratio between the necrosis and total tumoral volumes) for supratentorial and infratentorial lesions.

In terms of their localization, for both GBM and BM lesions, only the *R*_N/T_ showed a *p*-value of 0.002 (Mann–Whitney-U test), with greater values for supratentorial lesions than for infratentorial ones (see Figure 7). This referenced ratio had a median value of 0.2, a minimum of 0 and maximum of 0.61 for the supratentorial sites and a median value of 0.03, with a minimum of 0 and maximum of 0.2 for the infratentorial ones.

**Figure 8:**
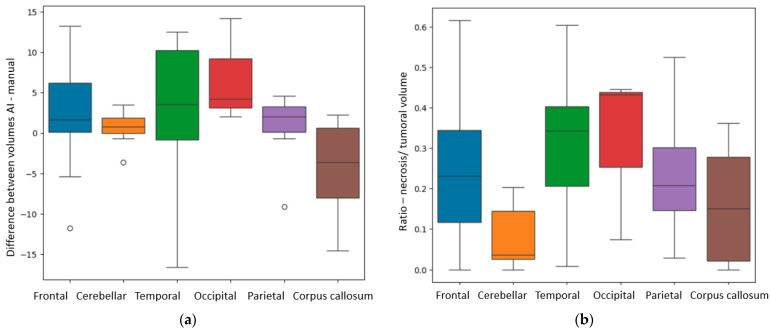
(**a**) Box plot showing the Diff _AI-M_ (difference in calculation of total tumoral volumes by AI and manual methods) depending on anatomical site. (**b**) Box plot showing the *R* _N/T_ (ratio between the necrosis and total tumoral volumes) depending on anatomical site.

Depending on the precise localization of the BM, we also found some significant results in relation to the difference (in cm^3^) between the volumes measured by the AI model and the manual method (Diff _AI-M_). We compared the volumes from different anatomical sites, both supra- and infratentorial: frontal, temporal, occipital and parietal lobes, the corpus callosum, all supratentorial and the cerebellum, infratentorial. The *p*-value calculated with the ANOVA one-way test was equal to 0.01. Figure 8a illustrates that, for lesions of the occipital and temporal lobes, the Diff _AI-M_ was greater than for other sites, with a median difference of 4.2 ± 6.51 cm^3^ and 3.5 ± 9.09 cm^3^, respectively. The Diff _AI-M_ was different depending on the lesions’ location, for the parietal lesions, the value was of 2.02 ± 4.63 cm^3^, for the frontal lesions, 1.63 ± 5.02 cm^3^, for the cerebellar site, 0.7 ± 1.94 cm^3^, and respectively for the corpus callosum 0.3 ± 6.11 cm^3^.

Using the Kruskal test, we also found a *p*-value of 0.006 for the *R*_N/T_ depending on specific distribution. The anatomical sites in which the tumors had a larger necrotic center were the occipital lobe, the temporal lobes and the frontal lobes, as seen in Figure 8b.

## 4. Discussion

Our findings provide compelling evidence of significant differences in the conventional MRI appearance of GBM and solitary BM. First of all, there is a quantitative difference in the degree of perilesional edema, with greater values for secondary lesions than for GBM (*p* = 0.04). This may be the result of the different dynamics in tumoral growth, since metastatic disease tends to progress slower than aggressive primary tumors such as GBM. Therefore, the volume of perilesional edema appears to be directly proportional to the duration of the secondary lesions’ evolution. Perilesional edema is the result of a chronic mass effect on impaired adjacent brain parenchyma, and it is seen on T2W and T2W FLAIR images as an area of hyperintensity [26].

Another important aspect that we would like to emphasize on is the difference between vascular edema and invasion of the surrounding parenchyma, usually found in primary aggressive tumors such as GBM [27]. GBM is less edematous and determines a rapid local invasion by infiltrating adjacent brain tissue along tracts of white matter, blood vessels and the subarachnoid space [27,28].

The distinction between brain invasion and perilesional edema is not always clear on conventional MRI, as they are both hyperintense on T2W and T2W FLAIR [26]. However, tumoral invasion can be seen as a diffuse area with diffusion restriction and no enhancement on contrast-enhanced MRI images. The presence of adjacent brain invasion alters the therapeutic protocol, shifting the focus to radiotherapy and leading to a greater risk of recurrence [16]. Numerous research papers have analyzed the peripheral edema of the brain lesions, especially for GBM, using functional and advanced MRI techniques, such as perfusion, diffusion, tractography and spectroscopy, with promising results [15,16,17]. Multiple studies found significant differences in the peritumoral edema of GBM and BM, in comparison with the intra-tumoral area, suggesting that future research should mainly focus on the peritumoral region [7].

Even though the differentiation between tumoral invasion and edema is a challenging task, the identification of surrounding brain parenchyma invasion by ML or DL programs can be performed using diffusion tensor imaging (DTI), an advanced MRI protocol that analyzes the extracellular water content, calculating the fractional anisotropy and the mean diffusivity [16].

DTI can be particularly useful in discriminating between the peritumoral edema of the metastasis and the peritumoral invasion of GBM, as in GBM water particles change their isotropic movement when destruction of white matter fibers occurs [6]. One particular research also studied the difference in the DTI signal of the tumoral lesions, with no discrepancy between the two. Instead, they found significant disparity in the peritumoral edema, with it being more heterogeneous in the proximity of GBM than BM, where the edema was predominantly or completely vasogenic in nature [6]. This suggests early parenchymal neoplastic invasion of GBM in the course of its development and the expansive nature of BM [7].

**Figure 9:**
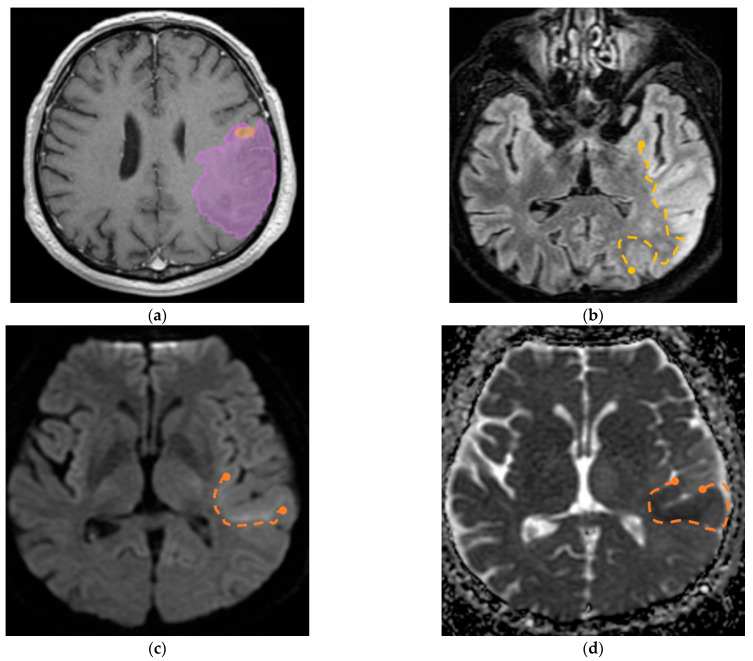
(**a**) Mdbrain processing of MRI brain acquisition (DICOM) (Scale 1:20)—purple area corresponding to T2W FLAIR hyperintensity; (**b**) T2W FLAIR (Scale 1:16), axial plane, manual delimitated (yellow dotted line) hyperintense area corresponding to edema/parenchymal invasion; (**c**) DWI (Scale 1:25), axial plane, discreet hyperintensity in the immediate subcortical area, manually delineated (orange dotted line); (**d**) ADC map (Scale 1:26)—hypo-intensity corresponding to the DWI hypersignal, suggesting parenchymal invasion, manually delimitated (orange dotted line) (approval was obtained from the Ethics Committee of the University of Medicine and Pharmacy “Grigore T. Popa” Iasi).

Furthermore, we identified a specific case involving a patient diagnosed with GBM with visible invasion in the adjacent parenchyma; the aspect on MRI imaging is seen in Figure 9.

Another finding strongly suggests that internal necrosis is more abundant in GBM lesions than in BM (*p* = 0.002). This could also emphasize the fact that GBM has a more aggressive nature, with greater cellular destruction and turnover. Consequently, the *R* _E/N_ was significantly greater for secondary brain lesions than for GBM (*p* = 0.02).

These findings conclude that GBMs have a more necrotic center and display less edema than BMs.

In clinical settings, the prompt identification and early differentiation of GBM and BM could significantly improve the patients’ overall survival, as suitable therapeutic protocols can be administrated, which is of particular benefit to patients with single BMs and undiagnosed primary tumors. In this case, the presence of a solitary brain lesion can mislead clinicians and radiologists into believing it could be a primary tumor of the Central Nervous System (CNS). Surgical resection would follow, and the search for the primary tumor and other systemic metastases would be delayed, beginning after the histopathological confirmation of the secondary lesion. In this scenario, not only is the proper treatment delayed, but the surgical resection could also interfere with the outcome of other local therapies as stereotactic radiotherapy.

The incidence of primary tumors that determined the BM was similar to that of the general population, with the predominance of lung, breast carcinomas and melanomas, displaying slightly disproportionate values due to selection bias [29]. Lung cancer was the most frequent neoplasia, representing in total 61.53% of cases, of which 53.84% were of the NSCLC subtype, followed by breast cancer (17.94%) and melanoma (7.69%). These results are not representative of the entire population, as they were obtained from a small cohort of patients, and should not be generalized.

The most edematous secondary lesions were from lung cancer, especially NSCLC, followed by digestive carcinomas, breast cancer and melanoma, with the least edematous being the BM from renal and colorectal carcinomas (*p* = 0.01). On the other hand, the most necrotic ones derived from colorectal and renal carcinomas (*p* = 0.01); this finding could be the result of scarce identification of cystic lesions by the AI model, as necrotic tissues and cystic transformation of lesions are quite similar on T2W FLAIR images.

**Figure 10:**
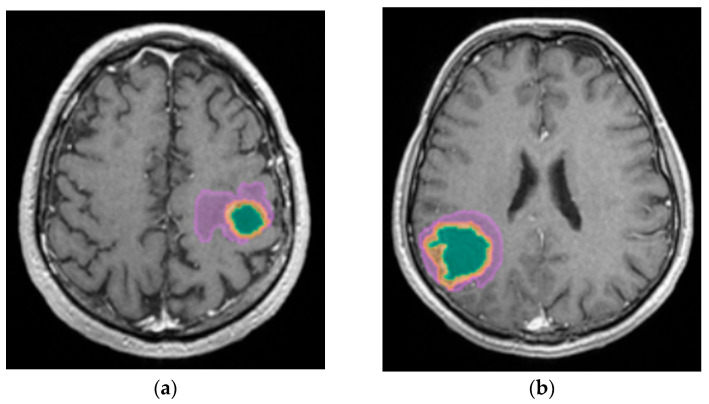
Mdbrain processing of MRI brain acquisition (DICOM). (**a**) T1W CE +FLAIR (Scale 1:24), axial plane, green area corresponding to the cystic portion of the lesion; (**b**) T1W CE + FLAIR (Scale 1:20), axial plane, green area corresponding to the necrotic center (approval was obtained from the Ethics Committee of the University of Medicine and Pharmacy “Grigore T. Popa” Iasi).

A better identification of cystic components and necrotic central tissue could be obtained by analyzing the contour defined by the lesion border, as necrotic borders are irregular and anfractuous, differing in thickness. In contrast, cystic walls are well delineated and smooth, having a more rounded shape (see Figure 10) [20].

Consequently, more studies should focus on this aspect, as their following implications are very different. Cystic lesions are radiotherapy resistant, often developing radiation necrosis, and their content is usually evacuated prior to other treatments. On the other hand, necrotic areas are surgically excised along with the tumor [30].

Regarding central necrosis, the most necrotic lesions were found in the supratentorial plane (*p* = 0.002), specifically in the occipital, temporal and frontal lobes (*p* = 0.006). The presence of central necrosis at diagnosis has been associated with a poor prognosis for patients with secondary lesions [31].

With reference to the accuracy of volume calculation by the ellipsoid manual and DL methods, the results are similar, with the greater difference being found among occipital and temporal lobe lesions (*p* = 0.01). This might be the consequence of the particular shape of the occipital and temporal lobes, as they are more distantly situated from the medio-sagittal plane, therefore, interfering in the ellipsoidal method of calculation, making it difficult to appreciate the true dimensions of lesions in these particular lobes. Another possible reason is the subjectivity of the manual method. Le Fèvre et al. compared two manual methods, the ellipsoidal method and the manual delineation of tumors, obtaining greater values for the ellipsoidal approach of tumoral volume calculation [19]. Our results show that the AI segmentation software obtained even greater values than the ellipsoidal method. Research comparing the three techniques might be useful in discovering which is the most accurate one. It is also important to take note that segmentation algorithms have similar accuracy in volume measurement, and none is more precise than any other [19].

Our study has its limitations as it is a retrospective, single-center research focusing on a relatively small cohort of patients (78 in total) diagnosed with GBM and BM. The results regarding the subtypes of BM should not be generalized due to the small sample size of many subgroups (one patient with single BM from renal carcinoma). We therefore strongly advise further correlations with other findings on this particular topic.

The retrospective nature of our study also brings a certain degree of diversity to the acquisition parameters that can affect the model results. The data collection was also retrospectively retrieved from the hospital’s registry of patients, and some data is consequently missing (for example, the molecular subtype of other primary tumors, such as breast cancer or melanoma). Although the inclusion and exclusion criteria helped build a solid database, consisting only of pathologically confirmed cases, this can also result in selection bias. This is why the incidence of GBM and BM in our study is not representative of the entire population.

Finally, further studies are required in order to better differentiate cystic lesions from central necroses, as well as perilesional edema from adjacent brain invasion in the case of aggressive tumors such as GBM.

## 5. Conclusions

To conclude, the DL software developed by mdbrain demonstrated its efficacy in the process of sub-segmenting brain lesions into solid and necrotic components, while also focusing on the perilesional edema. This provides greater information about the volume of both the lesion and the edema. Segmentation is an indispensable step in radiomics, preceding the feature extraction, and used alone, it can also provide relevant data regarding the tumoral burden and differentiation.

These applications could ease the crucial work of radiologists in the early identification of primary and secondary solitary lesions, with important results in time management and better decision-making for neurologists and neurosurgeons.

Moreover, our results confirm that GBMs are less edematous but more necrotic than secondary brain lesions.

More studies are needed in order to explore the morphological differences of GBM and BM, preferably using both conventional and advanced MRI sequences on a larger cohort of patients, with the inclusion of AI segmentation software.

## Figures and Tables

**Table 1:** Patients in the two groups—by sex.

Diagnosis	Women	Men
Glioblastoma	11	28
Brain metastasis	13	26

**Table 2:** Type of primary tumor.

Primary Tumor	Number (%)
Lung cancer (61.53%)	21 (NSCLC *) (53.85%)
	1 (SCLC **) (2.56%)
	2 (anaplastic) (5.13%)
Breast cancer	7 (17.95%)
Melanoma	3 (7.69%)
Colorectal	2 (5.13%)
Digestive (other sites)	2 (5.13%)
Renal	1 (2.56%)

* Non-small-cell lung carcinoma; ** small cell lung carcinoma.

## Data Availability

The data presented in this study are available on request from the corresponding author due to ethical restrictions.

## Abbreviations

The following abbreviations are used in this manuscript:

GBM	Glioblastoma
BM	Brain Metastasis
CT	Computed Tomography
MRI	Magnetic Resonance Imaging
ML	Machine Learning
DL	Deep Learning
AI	Artificial Intelligence
T2W FLAIR	T2-Weighted Fluid-Attenuated Inversion Recovery
CE T1W	Contrast-Enhanced T1-Weighted
FSPGR	Fast Spoiled Gradient Echo
DICOM	Digital Imaging and Communication in Medicine
NSCLC	Non-Small-Cell Lung Carcinoma
SCLC	Small Cell Lung Carcinoma
LC	Lung Cancer
DWI	Diffusion-Weighted Imaging
ADC	Apparent Diffusion Coefficient
DTI	Diffusion Tensor Imaging
CNS	Central Nervous System
